# Mobile phone–based interventions for mental health show promise of effectiveness, but what does the evidence tell us about what needs to come next?

**DOI:** 10.1371/journal.pdig.0000126

**Published:** 2022-11-03

**Authors:** Nicholas C. Jacobson, Patricia Areán, Stephen M. Schueller

**Affiliations:** 1 Center for Technology and Behavioral Health, Geisel School of Medicine, Dartmouth College, Lebanon, New Hampshire, United States of America; 2 Department of Biomedical Data Science, Geisel School of Medicine, Dartmouth College, Lebanon, New Hampshire, United States of America; 3 Department of Psychiatry, Geisel School of Medicine, Dartmouth College, Lebanon, New Hampshire, United States of America; 4 Department of Computer Science, Geisel School of Medicine, Dartmouth College, Hanover, New Hampshire, United States of America; 5 Department of Psychiatry and Behavioral Sciences, University of Washington, Seattle, Washington, United States of America; 6 University of Washington CREATIV Team, Seattle, Washington, United States of America; 7 Department of Psychological Science, University of California, Irvine, United States of America; 8 Department of Informatics, University of California, Irvine, Irvine, California, United States of America; The University of Sydney, AUSTRALIA

## Abstract

The current manuscript is a commentary on “Mobile phone–based interventions for mental health: A systematic meta-review of 14 meta-analyses of randomized controlled trials”. Although embedded within a nuanced discussion, one of the primary conclusions readers have taken from the meta-analysis was “we failed to find convincing evidence in support of any mobile phone–based intervention on any outcome”, which seems to contradict the entirety of the evidence presented when taken out of context of the methods applied. In evaluating whether the area produced “convincing evidence of efficacy,” the authors used a standard that appeared destined to fail. Specifically, the authors required “no evidence of publication bias”, which is a standard that would be unlikely to be found in any area of psychology or medicine. Second, the authors required low to moderate heterogeneity in effect sizes when comparing interventions with fundamentally different and entirely dissimilar target mechanisms. However absent these 2 untenable criteria, the authors actually found highly suggestive evidence of efficacy (*N* > 1,000, *p* < .000001) in (1) anxiety; (2) depression; (3) smoking cessation; (4) stress; and (5) quality of life. Perhaps the appropriate conclusions would be that existing syntheses of data testing smartphone intervention suggests that these interventions are promising, but additional work is needed to separate what types of interventions and mechanisms are more promising. Evidence syntheses will be useful as the field matures, but such syntheses should focus on smartphone treatments that are created equal (i.e., similar intent, features, goals, and linkages in a continuum of care model) or use standards for evidence that promote rigorous evaluation while allowing identification of resources that can help those in need.

## Mobile phone–based interventions for mental health show promise of effectiveness, but what does the evidence tell us about what needs to come next?

A recent publication in *PLOS Digital Health*, “Mobile phone–based interventions for mental health: A systematic meta-review of 14 meta-analysis of randomized controlled trials” [1], has received considerable attention, leading some to claim that mental health apps do not work because the science is flawed or the data is spare [2,3]. While most understand these are hyperbolic misinterpretations of the authors conclusions, the authors conclusions were “… we failed to find convincing evidence in support of any mobile phone–based intervention on any outcome”. We feel it is important to put this manuscript and the authors’ conclusion into context and to point out 2 fundamental problems with the methods by which the authors came to this conclusion.

The authors decided to set an extremely high bar to say whether or not apps are effective for the conditions they review. To illustrate, in order to establish “convincing evidence of efficacy”, 4 conditions needed to be met:

1. Total # of subjects (*N*) > 1,000
2. *p* < .000001
3. no evidence of publication bias
4. low to moderate heterogeneity in the effect sizes (I^2^ < 50%)

Based on these stringent criteria, the authors could have said in advance of their review that nothing would be “convincing”. Specifically, the last 2 criteria are most problematic and we will address each in turn beginning with the low to moderate heterogeneity and then no evidence of publication bias.

First, the authors set the assumption that all apps are created equal. Specifically, by requiring low to moderate heterogeneity in effect size (I^2^ < 50%) to establish “convincing evidence of efficacy”, this requires all studies to have an approximately similar level of efficacy when the app’s purpose, target mechanisms, and, thus, the study design are very different.

Indeed, an app in this study could technically include any type of intervention as long as it was delivered via an app or text message. For instance, the primary studies included in this meta-analysis included serious games for depression [4], which included neuropsychological training paradigms delivered through a video game interface (and was early in its development), and assumed this was be considered equivalent to interventions that included message-based care, which included of conducting evidence-based psychotherapies with a licensed clinician through secure messaging [5]. These interventions do not have the same mechanistic action nor are they on par with each other as to the therapeutic element. To put it simply, the meta-analysis included many types of apps that vary in their type of strategies, i.e., assessments and mood trackers [6], chatbots, meditation apps [7], serious games [4], as well as their type of care—self-guided, human-supported, and virtual care platforms [8]. Just because all these can be delivered through apps from mood trackers, in-the-moment assessment and brief intervention, serious games, message-based care, and meditation apps does not mean they should be lumped together; instead, it is best to combine and compare similar things to each other. In essence, the authors compared apples and oranges and concluded that neither were viable food because they both did not taste the same.

Second, the requirement to demonstrate “no evidence of publication bias” seems arbitrarily high, given that at least weak publication bias seems to occur in most areas of psychology and medicine [9], and in our read of umbrella reviews, this is not a necessary, nor common, requirement, one only need document the degree to which there is bias [10]. Very few interventions would meet all the above criteria, and, therefore, their conclusions follow more from the methods than the data.

Moreover, the use of an umbrella review may be premature, as the field is still growing and adapting, and such reviews are generally reserved for situations when there are many evidence-based options for one condition. For instance, to date, we still do not fully understand what optimal app engagement should be [11], given that apps are not like traditional treatment and can be used as often as needed.

Given these 2 problems, we offer a different interpretation of their findings. In the absence of these 2 very high bars, and if one inspects their density and forest plots, practically every study shows evidence of effectiveness in the following domains evidence of efficacy (*N* > 1,000, *p* < .000001), anxiety, depression, smoking cessation, stress, quality of life, findings consistent with recommendations from the Banbury Forum [12]. The appropriate conclusion should have been “Although more work is warranted, nearly all existing published data looks promising.”

So what do we learn from this study and where does the field need to go next? First, the evidence is promising for a number of common mental and behavioral health concerns. Second, not all apps are created equal; these tools have different features, goals, and linkages in a continuum of care model, it would be best to stop lumping them together, but rather make better distinctions between them. Third, we need more evidence, but we also need to set reasonable standards to ensure ineffective resources are designated as such, but not to set them too high, as this will prevent people from accessing potentially useful services.
